# Machine learning insights into vaccine adjuvants and immune outcomes

**DOI:** 10.3389/fimmu.2025.1654060

**Published:** 2025-10-07

**Authors:** Yuhyun Ji, Kavitha Bekkari, Ruchin Patel, Mohammed Shardar, Geoffrey A. Walford, SamMoon Kim, Yaping Liu, Willis Read-Button, Kristina Tracy, Jennifer Kriss, Colleen Barr, Marissa Wolfle, Shailaa Kummar, Celia LaPorta, Madison Radnoff, Milan Ghodasara, Jian Xiong, William J. Smith, Kunal Bakshi, Nicole L. Sullivan, Nicholas Murgolo

**Affiliations:** Merck & Co., Inc., Rahway, NJ, United States

**Keywords:** machine learning, deep learning, artificial intelligence, adjuvant, vaccine, RNA transcriptomics, antibody titers, immune response

## Abstract

Adjuvants boost the immune response to vaccine antigens, serving as key components in safe and effective vaccines. However, selecting a suitable adjuvant for a new vaccine can be challenging. This is due to the wide variety of adjuvants and the many mechanisms of vaccines they are meant to enhance. Therefore, the adjuvant selection process heavily relies on empirical experiments, which are time-consuming and resource-intensive. In this study, we introduce a machine learning approach leveraging non-human primate RNA transcriptomic data to predict immunogenic antibody levels after vaccination. Furthermore, analysis of the trained deep learning models enabled the identification of immune response mechanisms that are stimulated by adjuvants. Integration of machine learning has the potential to expedite vaccine adjuvant selection by focusing on evaluating adjuvant candidates with the highest probability of success. This may ultimately facilitate the development of more effective vaccines.

## 1 Introduction

Adjuvants are immune-boosting substances that are added to vaccines to enhance their immunogenicity and stimulate a more robust and long-lasting immune response (1–3). The use of adjuvants in vaccines has played a significant role in enhancing vaccine efficacy, reducing the required antigen dose, and improving the overall effectiveness of immunization programs (4–6). Among the different adjuvants that have been developed and utilized in vaccines, alum, monophosphoryl lipid A (MPL), and immunostimulating complexes (ISCOMs) are popular and have shown promising results in boosting immune responses (1, 3, 7). The primary aspects of adjuvants are generally categorized into two modes of action: enhancing the innate immune response and modulating the adaptive immune response (8–11). Adjuvants are known to activate and recruit various cells of the innate immune system, thus priming the adaptive immune system (1–3). However, there are variations in the specific mechanisms by which different adjuvants achieve these effects (12–14). Therefore, it is challenging to determine a single mode of action applicable to all adjuvants universally, and understanding how they alter or enhance vaccine-induced immune responses is often unclear (2, 13, 15).

Vaccine design heavily relies on an empirically intensive process due to the complex mechanisms and multifactorial nature of how adjuvants enhance vaccine-induced immune responses (9, 11, 16). Adjuvants can have varying effects in shaping the immune response when used in vaccines, and their effects are influenced by several factors (3, 12, 13, 17). For instance, the characteristics of antigens interact with adjuvants in their unique ways, potentially altering the elicited immune response (18–20). The choice of vaccine formulation and delivery systems can also impact the immune cell targeting, antigen uptake, and subsequent immune signaling events (3, 21, 22). Additionally, host factors, such as age, sex, smoking, and previous infections, can impact the immune response (23, 24). In consequence, researchers typically evaluate various adjuvants in combination with different antigens to identify the most effective adjuvant for a particular target (25, 26). This labor- and resource-intensive process hampers vaccine development, consequently hindering the widespread use of vaccines in a timely manner.

To overcome the challenges and streamline antigen/adjuvant vaccine design, we introduced a machine learning approach for predicting the efficacy of adjuvants by taking into account the early RNA signatures that interplay among various immune cells, cytokines, and signaling pathways. By utilizing systems biology, molecular modeling, and bioinformatics tools, researchers have tried to assist the vaccine development process to efficiently achieve the desired immune outcomes (21, 27–29). However, progress from empirical experiments to computational approaches has been impeded because of suboptimal performances of statistics-based computational tools for processing complicated immune data (21, 27, 28, 30). To model complex patterns and non-linear relationships that might not be apparent to human or statistical methods, machine learning algorithms are being actively incorporated in several stages of vaccine design (15, 20, 31–35). Particularly, our study mainly aims to integrate machine learning models to predict vaccine efficacy in an omics data aspect, which can contribute across the vaccine development phases, leading to more comprehensive computational tools.

Our study demonstrates two main results: 1. the superior performance of deep learning compared to traditional statistical tools, and 2. the potential of deep learning to predict the efficacy of adjuvanted vaccines. First, we began by applying artificial intelligence (AI) to the classification task, as distinguishing between different adjuvant groups based on RNA expression data is generally more straightforward and serves as an initial test of model feasibility. By comparing a statistical learning method (random forest model) with a deep learning approach, we established that deep learning models could more effectively identify group-specific transcriptomic patterns induced by various adjuvants. Second, building on this foundation, we then addressed the more complex challenge of predicting vaccine efficacy—specifically, antibody responses to adjuvanted 9-valent HPV vaccine—using early-stage RNA expression profiles. This stepwise approach allowed us to systematically evaluate the strengths of AI in both classification and regression tasks within transcriptomic analysis. Furthermore, by analyzing the weights of the trained deep learning networks, we identified gene sets that differentiate adjuvant-induced RNA patterns, providing mechanistic insights into adjuvant action. Collectively, our findings highlight the promise of deep learning not only for accurately classifying adjuvant types but also for predicting immune responses, thereby supporting its potential to accelerate and refine adjuvant selection in vaccine development.

## 2 Materials and methods

### 2.1 Preparation of HPV VLP vaccine with adjuvants

The 9-valent HPV vaccine was prepared using the major capsid protein L1 of the human papillomavirus (HPV) (36–39). Briefly, virus-like particles (VLPs) with recombinant HPV L1 major capsid protein were independently produced intracellularly in a Saccharomyces cerevisiae expression system. The cells were harvested and lysed, and the self-assembled L1 protein VLPs were purified chromatographically. The purified L1 protein VLPs morphologically resembled the authentic HPV virions but contained no viral DNA. Subsequently, VLPs were allowed for post-purification disassembly and reassembly treatment during bioprocessing to improve VLP immunoreactivity and stability (22, 40). The HPV VLPs were absorbed to aluminum hydroxyphosphate sulfate (AAHS or alum) and subsequently blended with additional adjuvants, as detailed in **Table 1**.

**Table 1:** Comparative summary of adjuvants used in the 9-valent HPV vaccine study: This table provides the variety of adjuvants used across all experimental groups, each characterized by a unique adjuvant treatment.

Group	Immunostimulatory type	Licensed product (company)	Reference
1	Alum	Gardasil (Merck)	(44, 45)
2	Alum + Lipid nanoparticle	Comirnaty (Pfizer), Spikevax (Moderna)	(46)
3	Alum + Chitosan nanoparticle	Not Applicable	(47)
4	Alum + Emulsion-based nanoparticle	Not Applicable	(2)
5	Alum + Squalene	Fluad flu vaccine (Seqirus)	(48)
6	Alum + MPL, QS21 (AS01 like)	Shingrix Zoster (GSK)	(49, 50)
7	Alum + MPL (AS04 like)	Cervarix 2vHPV (GSK)	(24, 51)
8	Alum + Cage-like nanoparticle (ISCOM)	COVID-19 Vaccine (Novavax)	(7)

The adjuvant groups, along with their immunostimulatory molecules and formulation types, are listed to offer a comprehensive overview of the experimental setup. As a note, all groups have 25 µg alum present with the 9-valent HPV vaccine.

### 2.2 Non-human primates study design

**Figure 1:**
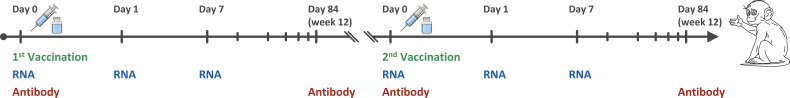
Schematic representation of the non-human primate (NHP) experimental design: This diagram illustrates the experimental design of our study involving NHP. Post-administration, blood was drawn at two intervals, 1 day and 7 days after the dose, to analyze the differentially expressed RNA levels. The RNA expression levels from two different time points were further analyzed to monitor the innate and adaptive immune response to the vaccine with adjuvant.

Non-human primates (NHPs) were on average 7.9 years old and weighed 9.86 kg at the study start (**Supplementary Table 1**). The male-to-female ratio of NHPs was about 4:1. The number of total NHPs used for this study was 60 and they were randomly assigned to 8 cohorts for vaccination. Information on randomization to a vaccine group is available in **Supplementary Table 1**. The adjuvanted vaccines (**Table 1**) were administered in a two-dose series; the first dose was given on day 0, and the second dose followed at week 24 (**Figure 1**). NHPs received a total intramuscular injection of 1.0 ml, divided equally with 0.5 ml administered over the right quadricep and 0.5 ml over the left quadriceps.

All studies utilized rhesus macaques (Macaca Mulatta) housed at the University of Louisiana at Lafayette, New Iberia Research Center (NIRC). Procedures involving the care and use of animals in the study were reviewed and approved by the Institutional Animal Care and Use Committee (IACUC) at both the Research Laboratories of Merck & Co., Inc., Rahway, NJ, USA and the University of Louisiana at Lafayette. During the study, the care and use of animals were conducted in accordance with the principles outlined in the guidance of the Association for Assessment and Accreditation of Laboratory Animal Care (AAALAC), the Animal Welfare Act, the American Veterinary Medical Association (AVMA) Panel on Euthanasia, and the Institute for Laboratory Animal Research (ILAR) Guide to the Care and Use of Laboratory Animals.

Prior to initiation, all animals underwent a physical examination by the study veterinarian or designate. The evaluation included a complete blood count, comprehensive chemistry analysis, and any other diagnostics requested by the study veterinarian to assess the health status of the animals. Only animals that, in the opinion of the study veterinarian, were healthy and otherwise met study criteria were admitted to the study.

Treatment of animals was done in accordance with NIRC standards. Prior to the vaccination and bleeds, the animals were sedated with ketamine hydrochloride, 10 mg/kg. Supplemental ketamine hydrochloride (5 mg/kg) was administered to maintain sedation for completion of all life activities. Administration routes include 2 × 0.5 mL (total of 1 mL per animal) of vaccine administered as an intramuscular injection dose per quadriceps muscle. Sedation agents were administered in an alternate site, as the quadriceps are the optimal vaccination site.

### 2.3 Immune profiling – RNA sequencing

Peripheral blood mononuclear cells (PBMCs) for mRNA profiles were collected to compare adjuvant effects on immune genes. Blood was collected at 6 different time points: pre-dose, 1 day after dose, and 7 days after dose (**Figure 1**). Total RNA was isolated from whole blood preserved in PAXgene blood RNA tubes. Total RNA was isolated using an accessory PAXgene blood RNA kit (PreAnalytix) according to the manufacturer's instructions. The quality and quantity of the total RNA sample were assessed using an Agilent Bioanalyzer with the RNA6000 Nano Lab Chip (Agilent Technologies).

For samples collected in PAXgene, cells were pelleted with centrifugation at 10,000 × g for 6 min. After the supernatant fluid was discarded, the cell pellets were washed by resuspension in 1 ml of dimethylpyrocarbonate (DMPC)-treated water, and then repelleted by centrifugation at 10,000 × g for 6 min. For all other media, cells were pelleted by centrifugation at 2000 × g for 30 min and used after removal of supernatant. All cell pellets were disrupted by vortexing in the lysis media appropriate for each extraction method.

Total RNA was extracted from samples using standard extraction methods ensuring high RNA integrity. RNA sequencing libraries were prepared using the TruSeq Stranded Total RNA Ribo-Zero kit (Illumina) according to the manufacturer’s instructions. This kit depletes ribosomal RNA to enrich for coding and non-coding transcripts, preserving strand specificity. Following RNA extraction and library preparation, the libraries were sequenced on an Illumina platform using a paired-end 50 base pair (bp) read format. Sequencing depth and multiplexing were adjusted according to experimental design to ensure sufficient coverage for downstream transcriptomic analyses (22, 23). Orthologues were identified as gene IDs listed in the Ensembl BioMart database.

Labeled cRNA was prepared by linear amplification of the Poly(A)+ RNA population within the total RNA sample. Total RNA was reverse transcribed after priming with a DNA oligonucleotide containing the T7 RNA polymerase promoter 5’ to a d(T)24 sequence. After second-strand cDNA synthesis and purification of double-stranded cDNA, *in vitro* transcription was performed using T7 RNA polymerase. The quantity and quality of the cRNA were assayed by spectrophotometry and on the Agilent Bioanalyzer as indicated for total RNA analysis.

Purified cRNA was fragmented to a uniform size and applied to Agilent Sheep 8x15K or Agilent Human 8x60K v2 Gene Expression microarray (Agilent Technologies, Sheep Design ID 019921, Human design ID 039494) in a hybridization buffer. Arrays were hybridized at 37°C for 18 hours in a rotating incubator, washed, and scanned with a G2565 Microarray Scanner (Agilent Technologies).

Arrays were processed with Agilent Feature Extraction software, and data was analyzed with GeneSpring GX software (Agilent Technologies). To compare individual expression values across arrays, raw intensity data from each gene was normalized to the 75th percentile intensity of each array. Genes were further normalized to the subject-specific PBS sample. Genes with values greater than background intensity in all replicates of at least one condition were filtered for further analysis (22, 23). Orthologues were identified as gene IDs listed in the Ensembl BioMart database.

### 2.4 Multiplexed meso scale discovery assay

To investigate adjuvant effects on adjuvanted vaccine immunogenicity in NHP, binding of the serum antibodies to the nine HPV VLP types was evaluated by multiplexed meso scale discovery (MSD) electrochemiluminescence assay. Customized 96-well MSD plates were pre-coated with 90 µg/ml of each of the nine HPV VLPs (6, 11, 16, 18, 31, 33, 45, 52, and 58) and stored at 4°C in individual sealed bags until analysis. MSD plates were removed from 4°C storage and allowed to come to room temperature (RT) prior to the assay. The plates were blocked with 150 µL of 3% nonfat milk in PBST with 0.05% Tween 20 with shaking at 400 rpm for 30 minutes at RT, then washed three times with 300 μL of PBST by a plate washer (BioTek).

NHP sera were diluted 1:100 in 1% fetal bovine serum (FBS)-PBST (Assay buffer) using an automated liquid handler (Agilent BRAVO). To calculate the antibody concentration in NHP sera, a reference standard cocktail was generated by pulling known concentrations of HPV-specific mouse monoclonal antibodies (mAbs) against each of the nine HPV types. The reference standard cocktail was serially diluted (5-fold) in the assay buffer to prepare a 7-point standard curve, and the assay buffer was used as blank well control (**Supplementary Figure 7**).

The reference standard and NHP serum sample dilutions were then added at 50 µl per well to MSD plates and incubated with shaking at 400rpm for 1 hour at RT. The plates were washed three times with 300 μL of PBST, then 50 µL of 0.5 ug/mL SULFO-TAG labeled goat anti-mouse or anti-NHP IgG (MSD) diluted in the assay buffer was added to wells containing mAb standard curve or NHP sera, respectively. After incubation with shaking at 400rpm for 1h at RT, the plates underwent a final three-wash with 300 μL of PBST. A total of 150 μL of MSD read buffer was then added per well and the plate was read on an MSD Meso Sector S600 instrument. Standard curves for the nine HPV type-specific mAbs were fitted using a 4-parameter logistic regression algorithm to calculate HPV type-specific antibody concentrations in the unknown samples (DISCOVERY WORKBENCH v4.0, MSD).

### 2.5 Data preparation for computational analysis and machine learning

**Figure 2:**
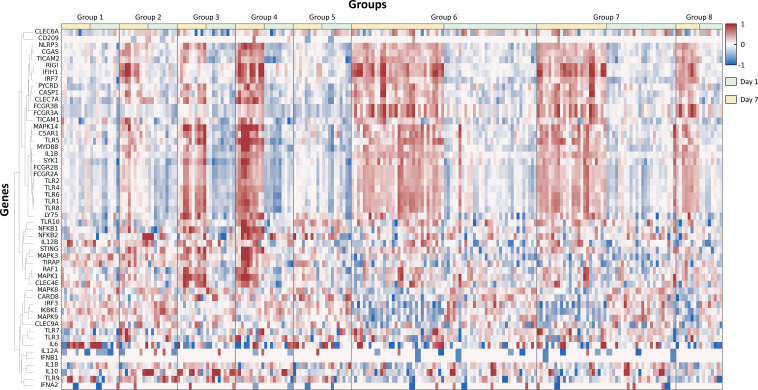
Hierarchically clustered heatmap of differentially expressed genes post-adjuvanted vaccine treatment: this figure presents a hierarchically clustered heatmap that visualizes the differential gene expression in groups treated with various adjuvants at two distinct time points, 1 day and 7 days post-dose. The dendrogram on the left shows the cluster linkage of genes, facilitating an easy comparison of gene expression across different treatments and time points.

**Figure 3:**
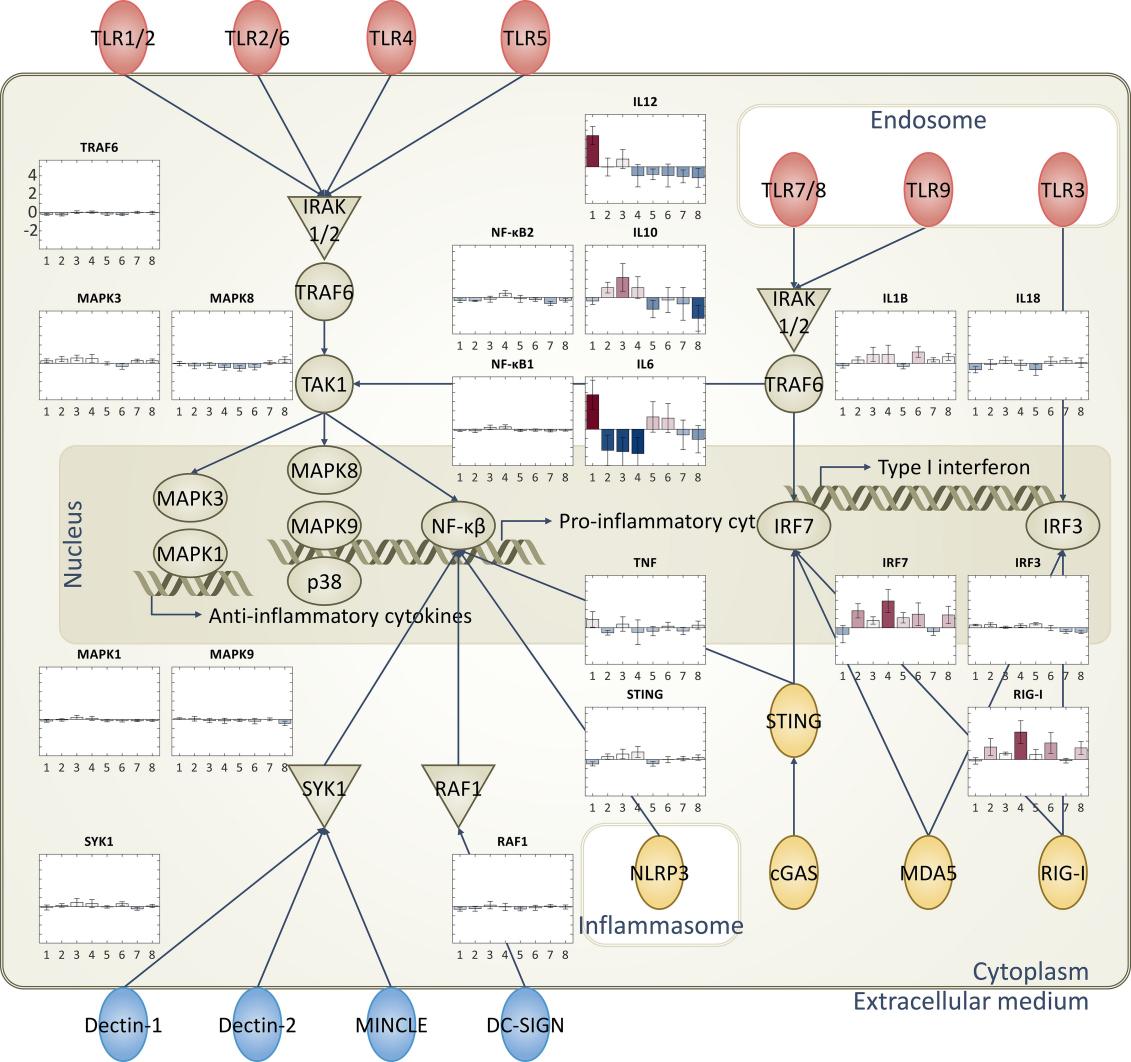
Diagrammatic representation of immune-related pathways and RNA expression levels: Immune response-related pathways and cytokines. Specifically focused on toll-like receptors (TLRs), cytosolic pattern recognition receptors, and C-type lectin receptors. Bar plots representing the RNA expression levels of each group are superimposed on these pathways, providing a comprehensive view of the immune response triggered by each adjuvant type.

RNA sequencing data was normalized to provide consistent and comparable measures of gene expression (mRNA expression) that can be used for performing expression analysis. Log 10-transformation was applied to fragments per kilobase of transcript per million mapped reads (FPKM) with a smoothing addon, pseudo count, of 0.01 for easier visualization and analysis. This small pseudo count (0.01) was to avoid division by zero, and the value was set small enough not to affect the results. Also, we used log10 to symmetrize up/down regulation. We filtered out transformed FPKM values (log(FPKM + 0.01)+0.01) that are below -1 to remove low-intensity data. Among a total number of 35,398 RNA sequencing data, 22,413 data showing low intensity were removed, and 12,985 data were left. Gene IDs of remaining RNA sequencing data were converted to the human equivalent using the BioMart-Ensemble database (https://mart.ensembl.org). 1,663 NHP genes that do not have human equivalents were ignored, and 11,322 gene data were left. We manually selected 1,184 immune-related genes from the remaining gene data for this study. Representative genes and cytokines related to immune-related pathways, including toll-like receptors (TLRs), cytosolic pattern recognition receptors, and C-type lectin receptors, are selected to show RNA expression patterns induced by different adjuvant types (**Figures 2**, **3**) (8–11).

### 2.6 Computational analysis – principal component analysis plot

The computational analysis was conducted to identify immunogenicity differences among different time points in the same cohorts and cohorts vaccinated with different adjuvants. Specifically, we carried out principal component analysis (PCA) to reduce the dimensionality of the dataset while retaining the most important information. This was done by transforming the original variables into a new set of uncorrelated variables known as principal components. The first principal component accounted for the largest possible variance, with each succeeding component accounting for the highest possible remaining variance. Using the PCA method, immune responses of 1 day and 7 days after vaccination were compared to their pre-immune reference point to show the differences between the two time points (**Supplementary Figure 1**). We used the StandardScaler data processing tool from the sklearn library in Python. Prior to downstream analyses of PCA, the input features were standardized to ensure comparability across variables. The StandardScaler transformation standardizes each feature independently by removing the mean and scaling to unit variance. This transformation centers the data around zero and rescales it so that each feature has a mean of zero and a standard deviation of one. By standardizing the features, we ensured that all variables contributed equally to the analysis, preventing those with inherently larger scales or variances from dominating the results.

### 2.7 Gene set enrichment analysis

We conducted a gene set enrichment analysis (GSEA) to explore the functional implications of the list of 1,184 immune-related genes selected specifically for our study. Leveraging the web-based tool Enrichr, we analyzed the biological context of these genes. Enrichr offers curated gene sets from well-established databases, including WikiPathways, Reactome, BioPlanet, and BioCarta. These gene sets represent distinct biological pathways, cellular components, and molecular functions. Our analysis involved assessing whether our ranked gene list demonstrated enrichment or depletion within these pathways, based on the associated p-values. To visually represent our findings, we generated enrichment plots. Additionally, we summarized the top 10 impacted pathways across the four databases using bar graphs (**Supplementary Figure 2**).

### 2.8 Machine learning design (deep learning/random forest)

Total RNA transcriptome data from animals were randomly split into 10 different folds for applying cross-validation. The cross-validation approach was considered to prevent problems that can be caused by the limited data, such as an insufficient amount of training dataset and class imbalance. During the 10 iterations of the training and testing process, 9 folds were used as a training dataset, and the remaining fold was used as a testing dataset. We conducted 10 iterations to cover all folds as testing datasets. The testing results from all 10 iterations were averaged to show the total accuracy of the entire dataset.

**Figure 4:**
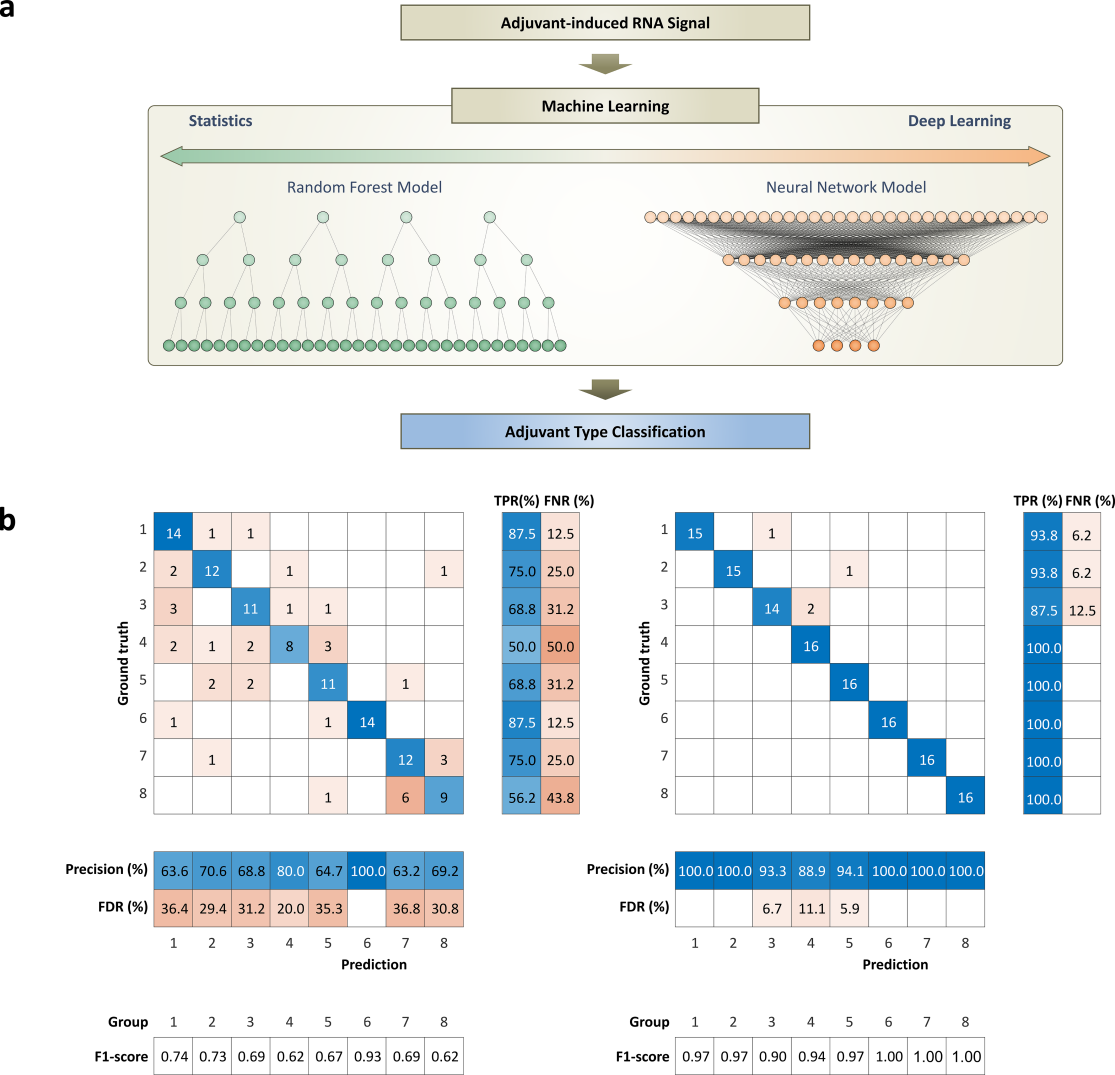
Working flow of machine learning models and performance for classifying adjuvant-induced RNA expression patterns: **(a)** Diagram of input and output for machine learning models, random forest and deep learning, used to classify RNA expression patterns induced by different adjuvants. **(b)** Performance evaluation and comparative analysis of machine learning models. The performances of the models are evaluated using a confusion matrix to show their ability for classifying the adjuvant-induced RNA expression patterns. Matric for indicating performances of each class includes true positive rate (TPR), false negative rate (FNR), precision, false discovery rate (FDR), and F1-score. These matrices provide a clear visual representation of each model’s classification performance, including true positives, false positives, true negatives, and false negatives. The total classification accuracies of the random forest model and deep learning model are 71.1% and 96.9%, respectively.

For the classification problem, we used two machine learning models, deep learning and random forest model, to see if machine learning could differentiate an adjuvant-specific immune profile (**Figure 4a**). The machine learning models for classification problems were fed immune responses as an input and adjuvant type as an output for training (**Figure 4a**). Briefly, the neural network was designed to have an input layer with 1,184 features, a hidden layer with 100 nodes, and an output layer corresponding to the classes to predict.

To address the issue of class imbalance in the training dataset, which can lead to biased model predictions favoring the majority classes, we implemented a data balancing strategy. Specifically, we ensured that each class contributed an equal number of samples during both the training and testing phases. This was achieved by undersampling the majority classes (groups 6 and 7) to match the size of the other classes. By maintaining a balanced dataset, the model was trained and evaluated on an equal representation of all classes, thereby mitigating bias and improving the fairness and robustness of the classification performance.

**Figure 5:**
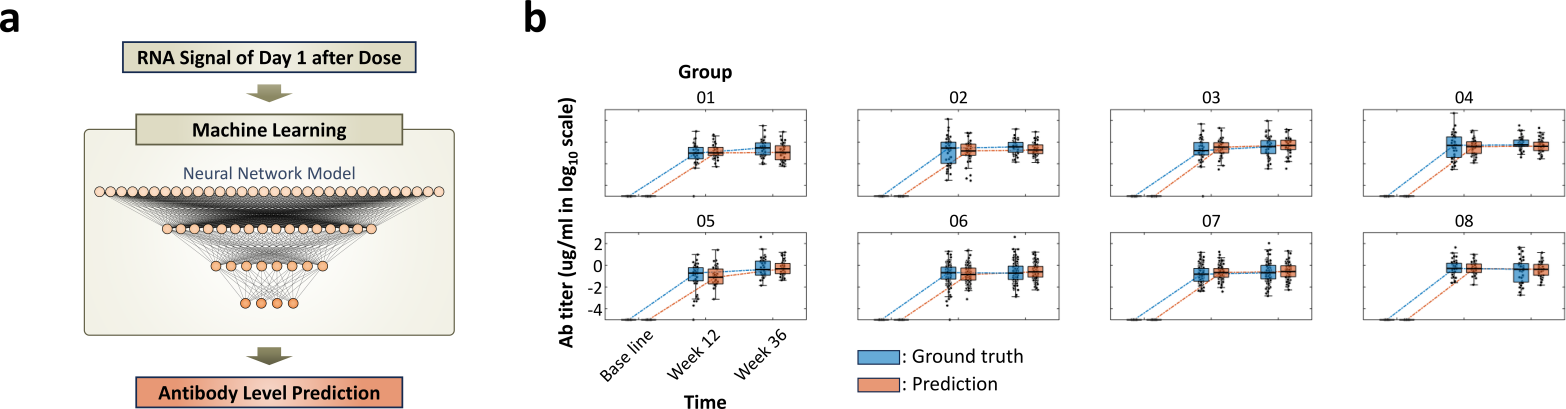
Working flow of deep learning model for predicting antibody titer and comparison with the ground truth value, measured antibody levels: **(a)** Diagram of input and output for deep learning model for predicting antibody titer. Information from day 1 after dose is used as an input to predict antibody levels of week 12 and 36. **(b)** Box-and-whisker plot illustrating the comparison between predicted antibody levels from deep learning model (orange box) and ground truth levels (blue box). The trends and mean antibody amounts between predictions and ground truth values show high alignment. The results demonstrate the capability of the deep learning model in understanding vaccine-induced RNA expression differences and the accuracy in predicting antibody levels. Total prediction results for the 8 groups with 9-valent are shown in **Supplementary Figure 6**.

For the immune response prediction problem, we used a deep learning model that takes innate (early-stage) immune response as an input and generates adaptive (late-stage) immune response and antibody titer as an output (**Figure 5a**). The network design was the same as the network for the classification task, but added a regression layer at the end to predict antibody levels of 9 HPV types. All the deep learning networks were designed to have fully connected networks to handle RNA expression data, and there were few reasons for selecting a fully connected network.

First of all, adjacent data points in RNA data do not have a strong relationship, and each data point can have its own information individually. In images or spectral data, data must be placed in a specific sequence to convey information. Images or spectral data may lose their meaningful interpretation when data points are misaligned, randomly shuffled, or when only a few isolated data points are selected without preserving their original context. However, RNA information does not have correlations between individual data points, and each data point has its own meaning. Therefore, the order of individual data points does not affect the overall meaning of the data.

The fully connected layer applies a linear transformation through a weights matrix to make every input element influence every output result without considering the alignment of input data. Convolutional neural network (CNN) handles input data with filters or kernels, and the filter uses adjacent elements for propagating calculation. Therefore, CNN is a mathematically reasonable way of extracting meaningful features from sequentially aligned data, such as images or spectra. Unlike CNN, the fully connected networks take all input data without considering their sequential order because every neuron in one layer is connected to every neuron in the other layer. This structural agnostic property and the calculating interpretation of the fully connected networks are suitable for handling data that does not have a specific order, such as RNA expression data.

### 2.9 Weights (saliency) analysis

The trained network with the highest predicted likelihoods for each adjuvant class was calculated independently for saliency analysis. The weights of each layer from the trained network are multiplied after considering the activation function, ReLU, to get a weight matrix per class, adjuvant group.

The input layer has 1,184 nodes, which is an equal number to the input size. The first hidden layer ( $L_{1}$ ) has 100 nodes. The $n^{th}$ node (0 < *n* < 101) in the first hidden layer connecting the $m^{th}$ node (0 < *m* < 1,185) in the input layer has a weight $W_{n,m}$ , after the training. The second hidden layer ( $L_{2}$ ) has 13 nodes, which is an equal number to the number of classes. The $p^{th}$ node (0 < p < 14) in the second hidden layer connecting the q^th^ node (0 < q < 101) in the first hidden layer has a weight $W_{p,q}$ . All the weights posed to the input data for the classification task can be calculated after taking the ReLU (Rectified Linear Unit) function into consideration. In this way, we were able to get the weights ( $W_{r,s}$ ) imposed on the r^th^ genes in the input layer for classifying the s^th^ adjuvant group. Since we used the cross-validation technique with 10 folds, there were 10 different trained models. Even though the training datasets were split into 10 folds and different sets of training data were given, each model stressed the weight of the genes in a similar pattern.

Notably, the ReLU activation function, defined as f(x) = max(0, x), which ensures that all output values are non-negative. During the saliency analysis, we calculated the overall importance of each gene by multiplying the weights across all layers of the trained deep learning network, taking into account the effect of the ReLU activation at each step. This approach inherently sets any negative values to zero, thereby focusing the analysis on genes that positively contribute to the model’s predictions. To efficiently organize the intensities per adjuvant group, we used a hierarchical clustering approach with a Correlation distance measure. Further, the intensity of weights imposed on each gene is visualized using a heat map, with arbitrary units normalized to a range from 0 to 1 as shown in the **Supplementary Figure 3**.

In this scale, a value of 0 represents the lowest weight, indicating minimal contribution, while a value of 1 corresponds to the highest weight, signifying maximal importance in the model. In other words, genes with weight values close to zero are interpreted as having little to no significance in describing the relationship between input features and output classes, whereas genes with weight values approaching one are considered highly important and sensitive indicators of the input-output relationship. This method allows for an intuitive and interpretable visualization of gene importance, facilitating the identification of key molecular features that drive the classification and prediction tasks within our study.

For further analysis and validation of genes showing high and low importance for describing the input and output relationship, we selected 100 genes that show high and low intensities across the adjuvant types. These selected gene sets were further analyzed statistically with the Pearson correlation coefficient, as shown in **Supplementary Figure 4**.

### 2.10 Data and code availability

The data that support the findings of this study are available, but restrictions apply to the availability of these data, which were used under license for the current study, and so are not publicly available. Data are, however, available from the corresponding author upon reasonable request and with permission of Merck & Co., Inc. The underlying code for this study is not publicly available but may be made available to qualified researchers on reasonable request from the corresponding author.

## 3 Results

### 3.1 Non-human primates study with 8 different adjuvants for HPV vaccine

In our study, we utilize 8 adjuvants, which have been previously employed in various vaccines, including those for HPV and influenza. Each adjuvant contains unique immunostimulatory molecules designed to enhance the immune response. The adjuvants are also formulated in different ways, including as lipid nanoparticles or liposomes. This experimental design contributes to understanding how different adjuvants and their formulations can influence RNA expressions and the performance of machine learning models.

NHPs were dosed with the 9-valent HPV vaccine plus different adjuvants, as detailed in **Table 1**. **Supplementary Table 1** lists the detailed information of the NHPs, such as age and weight, used in this study. Post-administration, blood was drawn at two intervals, 1 day and 7 days after the dose, to analyze the differentially expressed RNA levels (**Figure 1**, **Supplementary Figure 1**). This allows us to monitor the role of various adjuvants in modulating innate (day 1) and adaptive (day 7) immune responses to the vaccine and adjuvant.

The database from the Ingenuity Pathway Analysis (IPA) program is used to select 1,184 genes among all RNA expression levels (Methods). Briefly, genes related to cellular immune response, humoral immune response, pathogen-influenced signaling, and cytokine signaling in the immune system are filtered for further analysis. Additionally, genes related to TLRs, pattern recognition receptors, and C-type lectin receptors are also included (**Figures 2**, **3**).

### 3.2 Hierarchical clustering analysis

The hierarchical clustering analysis is to navigate the complex terrain of RNA expression levels across adjuvant groups (**Figure 2**). This robust visualization technique illuminated the patterns and disparities in gene expression profiles, thereby elucidating the influence of vaccine adjuvants. The data was compared from day 1 and day 7 with the baseline data from day 0 (pre-dose) to adjust for subject-specific baselines and to show fold change values. From the 1,184 genes related to immune responses, 54 representative genes related to adjuvant response and pathogen recognition systems were selected to enhance the clarity of the heatmap presented in **Figure 2**. Also, hierarchical clustering analysis is shown in the heatmap plot for the comparison of differentially expressed RNA patterns.

The heatmap reveals unique fingerprints attributed to different adjuvant types. The heat map encapsulates the dynamic interaction of immune-related genes, unveiling clusters of co-expressed genes. The data from day 1 and day 7 show distinct differences, indicating that each adjuvant induces different innate and adaptive immune responses (**Supplementary Figure 1**). Additionally, different adjuvants induced unique fingerprints of immune responses, generating distinctive RNA expression patterns. Notably, groups 6 and 7, which both have MPL as an immune stimulatory molecule, show similar patterns.

### 3.3 Principal component analysis

As depicted in **Figure 2**, the two different time points, 1 day post-dose and 7 days post-dose, clearly exhibit different patterns due to their distinct innate and adaptive immune responses. To further support these differences, a PCA plot was generated. The principal components of the data from day 1 and day 7 are distinguishable, indicating that these two time points have different expression patterns (**Supplementary Figure 1**). This analysis provides valuable insights into the temporal dynamics of gene expression following adjuvant treatment, contributing to our understanding of the immune response dynamics.

### 3.4 Pathway analysis

Pathway maps were utilized to illustrate the distinct expression levels across different groups that are treated with various vaccine adjuvants. Different adjuvants induced unique patterns of gene expression in each group, particularly in pathways related to pathogen-influenced signaling. To visualize these differences, pathways, such as TLRs, pattern recognition receptors, and C-type lectin receptors, were evaluated since those pathways are integral to pathogen recognition steps. These specific pathways are known to play a crucial role in the immune response and are significantly influenced by the adjuvants evaluated in this study (**Figure 3**, **Supplementary Figure 2**).

Next, evaluation of a series of diagrams to visualize these differences was done with each representing a specific pathway (**Figure 3**). The expression levels of the elements in the pathways are visualized using thermometer bars, providing a clear and intuitive representation of the data. All the batches in the same group are averaged to show a representative value in the bar plot, along with the standard deviation. Bar plots on the side of each pathway allow us to easily compare the adjuvant-induced expression levels across different pathways and groups.

The analyses reveal that the vaccine adjuvants had a significant impact on the expression levels in the immune response-related pathways, especially in the pathogen recognition ligands. Notably, adjuvants with the same immunostimulatory molecule tend to show similar trends. For instance, groups 2 and 4, which contain the same lipid substance, both show high expression levels in IRF7 (transcription factor that drives production of antiviral interferons) and RIG-I (Viral RNA sensor that triggers antiviral signaling pathways) (41). Overall, all adjuvants show downregulation of the inflammatory response, which aligns well with previously known knowledge (**Figure 3**) (1).

As previously explored, each adjuvant exhibits a different mode of action, resulting in unique expression levels of ligands (1, 2). Despite the distinct RNA expression levels of ligands observed in different groups, there are similarities in the expression levels of certain cytokines at the end level of signaling pathways. The specific effects vary depending on the type of adjuvant used, underscoring the complexity of the immune response and the role of adjuvants in modulating this response. Because of the complexity of the signaling pathways, our understanding of these processes is not yet complete. However, this highlights the significance of integrating machine learning approaches to aid in understanding adjuvants for facilitating vaccine development.

### 3.5 Gene set enrichment analysis

Enrichment analyses play a pivotal role in bioinformatics, aiding researchers in interpreting complex genomic data. Among these methods, gene set analysis stands out as a valuable and widely adopted approach. In our study, GSEA was employed. GSEA is a computational method designed to identify gene sets that exhibit significant enrichment or depletion within a large gene pool.

Gene sets represent functionally related genes, each set reflecting a common biological theme. These themes can range from disease associations and chromosomal locations to regulatory pathways. In our investigation, gene sets were harnessed to represent specific biological pathways. To unravel the functional implications of our gene list, two web-based tools: Enrichr (**Supplementary Figures 2a–d**) and IPA were utilized (**Supplementary Figure 2e**). Enrichr and IPA function as gene signature search engines, capable of extending enrichment analysis while also facilitating downstream signal interpretation and functional analytics.

Enrichr, in particular, integrates more than 30 gene-set libraries, providing a comprehensive resource for pathway exploration. The interactive visualization approaches of Enrichr, powered by the JavaScript library Data-Driven Documents, offer a concise summary of known pathways based on a collective gene function list. These results from Enrichr provide a detailed overview of the enriched pathways and biological processes associated with the gene list that we selected as described in the methods.

IPA, on the other hand, transforms our gene list into a set of relevant networks based on extensive records maintained in the Ingenuity Pathways Knowledge Base (IPKB). This allows for the analysis and visualization of the data, providing insights into the biological context of the expression analyses. These IPA results highlight the key pathways and networks that our gene list is involved in, offering a deeper understanding of the biological implications of our findings.

Since the list we curated specifically focuses on immune response-related genes, the results of GSEA, as depicted in **Supplementary Figure 2**, reveal that the selected gene sets are highly related to antigen processing and presentation mechanisms. The analysis results from these databases reveal that the gene sets are highly related to antigen processing and presentation mechanisms. This includes Th1 activation, Th2 activation, TLRs, and C-type lectin receptors pathways.

**Supplementary Figure 2** further illustrates that these enriched pathways are not only statistically significant but also biologically meaningful in the context of adjuvant action. For example, the prominent enrichment of TLRs and C-type lectin receptor pathways highlights the role of adjuvants in activating innate immune sensors, which are crucial for initiating and shaping the adaptive immune response (1, 2). The activation of different pathways, as shown in **Figure 2**, reflects the ability of different adjuvants to skew the immune response toward either cellular or humoral immunity, depending on their mode of action. This mechanistic insight is particularly important as not only the balance between Th1 and Th2 responses, but also the pathogen recognition receptor pathways, can influence the overall efficacy and safety profile of a vaccine.

Furthermore, different adjuvants are known to enhance and shape the immune response to antigens through diverse mechanisms, ultimately aiming to influence both the efficacy and durability of vaccine-induced protection. Overall, aluminum promotes antigen uptake by antigen-presenting cells and induces strong Th2-type humoral responses, supporting robust antibody production. It is also known to stimulate the production of pro-inflammatory cytokines, such as IL-6, which is well aligned with the result in **Figure 3**. Lipid nanoparticles (LNP) (group 2) facilitate efficient delivery of nucleic acids and activate innate immune pathways, including TLRs, thereby promoting both cellular and humoral immunity. Previous work has shown that empty LNPs (no nucleic acid) can act as an adjuvant as well (42). Chitosan (group 3), a natural polysaccharide, enhances antigen uptake at mucosal surfaces and stimulates both Th1 and Th2 responses by activating pattern recognition receptors, such as TLRs and C-type lectin receptors. Chitosan, as a vaccine adjuvant, induces the production of IL-10, an anti-inflammatory cytokine, thereby helping to balance immune activation with the regulation of inflammation, as shown in **Figure 3**. Squalene (group 5), often formulated as an oil-in-water emulsion, recruits immune cells to the injection site and promotes antigen presentation, leading to a balanced Th1/Th2 response. MPL (groups 6 and 7), a TLR4 agonist, stimulates robust innate immune activation and skews the adaptive response toward Th1-type cellular immunity, which is crucial for protection against intracellular pathogens.

These findings provide valuable insights into the immune response mechanisms triggered by different adjuvants and contribute to our understanding of vaccine efficacy. By engaging distinct molecular pathways, adjuvants not only enhance the magnitude and quality of the immune response but also promote the development of immunological memory, resulting in more effective and longer-lasting vaccine protection. The genes identified in this study align well with previous knowledge and effectively represent the signals induced by the adjuvants. This not only validates the biological relevance of the gene selected in this study, but also offers a biological foundation and legitimacy of AI approaches for predicting the immunogenic potential of novel adjuvants based on their transcriptomic impact. Also, these tools provide valuable context for understanding the functional significance of the selected genes. This alignment validates the selection of genes and underscores the relevance of our study.

### 3.6 Machine learning models

Random forest models, while commonly and traditionally used for classification tasks, can struggle with complex datasets, such as those in bioinformatics (43). They are prone to overfitting, particularly when the data is noisy or high-dimensional. This sensitivity to noise can lead to poor performance on unseen data and difficulty distinguishing between relevant and irrelevant features. Furthermore, if the relationship between the features and the target variable is complex and non-linear, the model may struggle to capture these relationships accurately.

They can be computationally expensive and slow to train, particularly when the dataset is large. This is because the model needs to construct and store multiple decision trees, each of which requires computational resources (27). The interpretation of these models can also be challenging due to the large number of decision trees. Each tree contributes to the final prediction, making it difficult to understand the role of individual features. Furthermore, random forest models may not work well with categorical variables and can generate biased results. These limitations are particularly pronounced in fields like bioinformatics, where datasets are often large, complex, and include many data points (27).

To address the challenges of statistical approaches, we propose the use of a deep learning model, designed to handle large amounts of data with complex inter-gene relationships. First of all, the model was designed with fully connected layers to mimic the structural characteristics of the random forest model (**Figure 4a**). Models with convolutional layers are a common strategy, but the calculation method is more explainable when handling data where adjacent data points have meaningful information. This is because it uses a convolutional layer with kernels that stride on data for calculating data points right next to each other.

Images are a common data format for the 2-dimensional convolutional network. The network is trainable when each pixel in the image is in the right order to show the object in that image. If the pixels in the image are randomly sorted without order, we cannot see the features of an object in the image, and the machine learning model cannot be trained. In the same way, spectral information can only be delivered when they are arranged in the right order in the frequency domain. Sequential information of wavelength is crucial when using a 1-dimensional convolutional layer for finding meaningful features.

However, RNA expression data are 1-dimensional data without an order. The n^th^ gene (1 ≤ n ≤ 1,184) in the data may not have any relationship with the n-1^th^ or n+1^th^ gene. Considering that a fully connected layer doesn’t count the order of the input data, as in the random forest model, and it propagates the calculation of all the nodes independently, makes the fully connected layer more explainable and reasonable for our goal.

### 3.7 Machine learning – RNA classification

Our study compares the performance of two machine learning models: random forest and deep learning, in classifying RNA expression patterns induced by different adjuvants. The deep learning model outperforms the random forest model, achieving an accuracy (sum of all true positive (TP)/total number of samples) of 96.9%, while the random forest model achieves an accuracy of 71.1%. The random forest model particularly struggles to classify groups 7 and 8 (**Figure 4b**). As shown in **Figure 2**, the expression patterns are very similar to those of groups 7 and 8, which might confuse the random forest model. On the other hand, the deep learning model efficiently identifies the subtle differences between the signatures induced by the different adjuvant types, even though they have similar RNA expression patterns (**Figures 2**, **4b**). This is reflected in its high accuracy, concluding that the deep learning model can find subtle but unique features of adjuvant-induced signals that the random forest model wasn’t able to catch.

In addition to the accuracy, the confusion matrix in **Figure 4b** provides several key metrics for evaluating the multi-class classification performance of two machine learning models. For a given class k, the true positive rate (TPR), or recall, measures the proportion of actual class k samples correctly identified and is computed as ${\text{TP}}_{\text{k}}÷\left({\text{TP}}_{\text{k}}+{\text{FN}}_{\text{k}}\right)$ , where ${\text{TP}}_{\text{k}}$ is the count of correctly predicted samples of class k and ${\text{FN}}_{\text{k}}$ is the count of class k samples misclassified as other classes. The false negative rate (FNR) complements this by representing the proportion of class k samples missed, calculated as ${\text{FN}}_{\text{k}}÷\left({\text{TP}}_{\text{k}}+{\text{FN}}_{\text{k}}\right)$ . Precision for class k indicates the accuracy of predictions labeled as class k and is given by ${\text{TP}}_{\text{k}}÷\left({\text{TP}}_{\text{k}}+{\text{FP}}_{\text{k}}\right)$ , where ${\text{FP}}_{\text{k}}$ counts samples incorrectly predicted as class k. Additionally, the false discovery rate (FDR), defined as ${\text{FP}}_{\text{k}}÷\left({\text{TP}}_{\text{k}}+{\text{FP}}_{\text{k}}\right)$ , reflects the proportion of incorrect predictions among all predictions for class k. Also, the F1 scores for class k are derived as $2\left({\text{Precision}}_{\text{k}}\times {\text{Recall}}_{\text{k}}\right)÷\left({\text{Precision}}_{\text{k}}+{\text{Recall}}_{\text{k}}\right)$ to show the harmonic mean of precision and TPR, balancing both.

It’s important to note that previous studies achieved over 90% accuracy with the random forest model, which used a mere 10 to 20 genes to classify 2 adjuvant groups (34, 35). In contrast, this study used a set of 1,184 genes to train machine learning models to classify 8 adjuvant groups. This complexity likely contributes to the lower accuracy of the random forest model. These findings clearly demonstrated that the statistical model struggles to process complex datasets or complex classification tasks, while the deep learning model excels in these areas.

### 3.8 Saliency analysis

Using saliency analysis, evaluation of the deep learning network was done to comprehend how the deep learning model operates. By illustrating the influences of various genes on adjuvant prediction, evaluation of which genes gained more attention during the training process was done. The weights of each node are calculated as described in Methods. This methodology enables the identification of genes that contribute significantly to the classification of specific adjuvant groups by examining the weights assigned to them.

Heat maps showing saliency analysis to investigate the predictions of a deep neural network for adjuvant type classification were generated. Deep learning networks are often considered to be “black boxes” that offer no way of figuring out what a network has learned or which part of an input to the network was responsible for the prediction of the network. To understand genes that are influenced more when differentiating the immune signatures induced by adjuvants, the weights of the trained network were visualized in **Supplementary Figure 3**. Using the saliency analysis, specific genes of input RNA transcriptomes that focused more when classifying the adjuvant types were identified.

All the weights from the trained model are arranged in a heatmap, from high to low (**Supplementary Figure 3**). The weights for classifying groups 1 to 8 exhibit a similar trend, albeit not identical. Furthermore, the 10 trained models from 10 different folds display very similar trends, indicating that the training process was effectively executed to highlight specific genes for the classification task. Despite the black-box nature of the deep learning process, the consistent trend in the weights clearly demonstrates that the process is not random. This finding underscores the reliability and robustness of our deep learning model in classifying adjuvant groups.

The weights of each gene are arranged from high to low, as shown in **Supplementary Figure 3**. The top 100 genes with high weights across all classes on average were selected for further analysis. The RNA expression levels of the 100 genes with high weights underwent a Pearson correlation coefficient analysis. This analysis aims to understand the correlation between two different groups. The Pearson correlation coefficient measures the correlation between two sets of data. If the Pearson correlation coefficient is closer to 1, this means the two data sets have a higher correlation and a similar RNA expression pattern. Also, 100 genes with low intensities are selected to compare the analysis results of 100 high-intensity genes. Groups showing a high correlation with a p-value lower than 0.05 are depicted in red, while others are in black.

The RNA expression level of the top 100 weighted genes generally has low coefficient values between different groups, indicating that the expression levels of these gene sets are not identical. In other words, these genes received high weights because they were unique to groups, making them an important marker for classifying the groups. However, the RNA expression level of the 100 genes with low weights shows high coefficient values with a p-value lower than 0.05. This result suggests that the expression pattern of these 100 genes is similar across the adjuvant group. In other words, these 100 genes received low weights because they appeared similar across the groups and were not very useful for classifying each group.

Additionally, the RNA expression levels of the 100 genes with high weights are more similar in the day 7 data compared to the day 1 data (**Supplementary Figures 4a, b**). This suggests that the signals induced by the adjuvant are more consistent in the adaptive immune response than in the innate immune response. This observation underscores the dynamic nature of the immune response and the distinct roles that these genes play at different stages in adjuvant-induced signal pathways.

Furthermore, the Pearson correlation coefficients among groups 6 to 8 are significantly higher than others for both the top 100 genes and the bottom 100 genes. This indicates that the immune response of groups 6 to 9 and their RNA expression levels are highly correlated in a linear way. The similarities in the immune responses and gene expression patterns of these groups appear to present a challenge for the classification task, indicating the complexity of the biological processes involved. This finding provides further explanation for the observed lower accuracy of the random forest model when classifying different adjuvant types (**Figure 4b**). **Figure 4b** interprets with slope between two different adjuvant groups. Linear relationship of genes between two different groups indicates that the genes with low weights have similar patterns regardless of the groups. This means that the RNA expression patterns are similar between groups, and the deep learning network didn’t put stress on these genes during the training process, since they are not useful for solving the classification task.

To further analyze the highly weighted genes and understand the pathways that can differentiate various adjuvants, genes were compared within sets of the database (**Supplementary Figure 5**). The gene sets that were highly focused during the training process are involved in the anti-inflammatory signaling pathway, pro-inflammatory and cytokine signaling in the immune system, as well as the GPCR ligand binding pathway.

These genes and results further illuminate the path for adjuvant mechanism studies, providing insights into which pathways require more focus. Such analysis was not possible with statistical approaches due to the complexity of the data and the lack of computational algorithms. However, our custom deep learning network demonstrates the potential to be used for the analysis of complex pathways and differential expression levels of genes. This approach opens up new avenues for understanding the mechanisms of different adjuvants and their effects on the immune response.

### 3.9 Machine learning – antibody prediction

Finally, the capabilities of a machine learning algorithm for predicting antibody levels following vaccination were investigated. The experimental design involves analyzing the antibody levels of a 9-valent HPV vaccine at two critical time points: 12 weeks after the first vaccine dose and 12 weeks after the second dose (administered 24 weeks apart). To ensure accurate comparisons, we subtracted the baseline antibody level (measured at day 0) from each data point.

For the antibody level prediction, the deep learning model was employed that leveraged RNA expression levels measured on day 1 after vaccination. Specifically, the model generates the antibody levels of 12 weeks after the dose as an output by using the RNA expression levels of 1 day after the dose as an input. The architecture of the network includes a fully connected layer, as the network was used for the classification task. However, instead of using the One-hot encoding layer for the classification task as described above, the regression layer at the end was used to predict the antibody level from the RNA expression data.

To assess the performance of the machine learning tool, we compare the machine learning results with the ground truth antibody levels (**Figure 5b**, **Supplementary Figure 6**). The machine learning algorithm demonstrates remarkable accuracy in predicting antibody levels. Side-by-side comparisons with ground truth data highlight the similarities and validate the effectiveness of our approach. Notably, the model successfully predicted both early-stage (12 weeks after the first dose) and late-stage (12 weeks after the second dose) antibody levels to show the antibody changing over time. This ability to anticipate immune responses at different time points is crucial for vaccine development.

The model not only effectively captures the differences in RNA expression induced by the first and second vaccine doses, but it also differentiates adjuvant-specific patterns (**Figure 4**), allowing precise prediction of antibody trends for each group (**Figure 5**, **Supplementary Figure 6**). These results underscore the potential of machine learning to expedite vaccine development. By computationally anticipating antibody titers, experimental time and resource costs could be significantly reduced.

## 4 Conclusions

The integration of machine learning techniques has emerged as a valuable tool in the development of vaccines. Moreover, the global spread of pandemics underscores the urgency of equipping computational methods for rapidly developing vaccines to avoid facing similar challenges in the future. Scientists have been focusing on AI for building protein structures and calculating their binding affinities with molecules. By utilizing machine learning, scientists have successfully demonstrated the ability of computational approaches to expedite the screening and development of novel adjuvant candidates, thereby accelerating the vaccine development process. However, the binding of a pathogen-derived ligand to pathogen recognition receptors is only the first step that triggers a series of immune signaling events leading to the activation of the body’s defense response. There remains a vast, untapped area, such as cytokines and signaling pathways, following pathogen and receptor binding.

This study has demonstrated the potential of using computational approaches to identify the intricate and complex immune-related signals induced by adjuvants. Given the complexity of RNA expression levels and the relationship between RNA and antibodies, creating a model that accurately represents this relationship seemed difficult at best. Nevertheless, this paper showed that RNA expression levels exhibit recognizable patterns by using deep learning. This pattern wasn’t clearly understood with a conventional statistical approach, random forest model, but our custom deep learning model showed 96.9% accuracy for classifying the RNA patterns. Moreover, the data used for analysis of this custom deep learning model has successfully predicted the antibody amount from the RNA expression level that follows the trends of the ground truth values.

This paper is the first to clearly demonstrate the possibilities of using AI to understand unexplored areas of immunology and vaccines. Ultimately, this pioneering study can fill the gap between adjuvants and drug efficacy for the fully computational vaccine development process. Additionally, insights were provided to identify important genes for future studies by analyzing the genes that showed high importance for predicting antibody levels. Continued research and innovation in this field will drive the advancement of adjuvant-based vaccines and reduce the cost for the screening process, leading to improved global health outcomes.

## Data Availability

The data presented in the study are deposited in the NCBI GEO repository, accession number GSE309668.

## Supplementary material

Supplementary Table 1 Information of non-human primates used for this study.

Supplementary Figure 1 Principal component analysis of gene expression post-dose: A principal component analysis (PCA) plot that illustrates the variance in gene expression between samples taken at two distinct time points: 1 day post-dose and 7 days post-dose. The PCA plot encapsulates the differences and similarities between the two time points, providing a visual representation of the variance in gene expression.

Supplementary Figure 2 Gene set enrichment analysis (GSEA) of adjuvant-related genes using Biomart and Ingenuity Pathway Analysis (IPA): The analysis provides insights into the biological pathways these genes are involved in, offering a comprehensive view of the immune response. GSEA with a different databank for understanding the genes influenced by the adjuvants. **(a)** WikiPathways 2019 human, **(b)** Reactome 2022, **(c)** BioPlanet 2019, **(d)** BioCarta 2016, and **(e)** IPA. Black dotted lines on the figure indicate the p-value of 0.05.

Supplementary Figure 3 Weight analysis of trained deep learning model: a comprehensive analysis of the weights derived from the trained deep learning model. It emphasizes that some genes played a more pivotal role during the training process, thereby shedding light on the model’s decision-making mechanism.

Supplementary Figure 4 Pearson correlation coefficient analysis of genes having high and low weights in the trained model: Pearson correlation coefficient analysis of the top 100 genes that were most and least influential during training. The analysis offers insights into the relationship between genes and their impact on the model’s decision making. **(a)** Genes obtained high weight during the training process for the classification task. Low coefficient values (0.35 with 0.13 standard deviation) with gentle slope between two different adjuvant groups are notable. This means that the same genes showed different expression levels. In other words, genes with high weights show different RNA expression patterns that can easily distinguish between two different groups. **(b)** Genes obtained low eight during the training process for the classification task show a high coefficient value (0.50 with 0.18 standard deviation).

Supplementary Figure 5 Gene set enrichment analysis (GSEA) of highly weighted genes: This figure showcases a gene set enrichment analysis of the top 100 genes that had higher weights for the classification task. The analysis offers insights into the biological pathways these genes are involved in, thereby providing a deeper understanding of the adjuvant-induced changes. Also, the analysis can provide potential genes that should be more focused for studying adjuvant-specific mechanisms. GSEA with different datasets, including **(a)** WikiPathways 2019 human, **(b)** Reactome 2022, **(c)** BioPlanet 2019, and **(d)** BioCarta 2016, are shown.

Supplementary Figure 6 Predicted antibody titer using a deep learning model and its comparison with the ground truth value, measured antibody levels: Box-and-whisker plot illustrating the comparison between predicted antibody levels from deep learning model (orange boxes) and ground truth levels (blue boxes). The trends and mean antibody amounts between predictions and ground truth values show high alignment. The results demonstrate the capability of the deep learning model in understanding vaccine-induced RNA expression differences and the accuracy in predicting antibody levels.

Supplementary Figure 7 Standard curves for nine virus-like particle (VLP) types, displaying the measured signal for all seven standards (Std 1–Std 7). All points within the validated dynamic range are shown. A broad dynamic range indicates a reliable and proportional signal response across the entire concentration series. The y-axis is shown on a logarithmic scale to illustrate the full range of signal intensities.
