# Supplementary material for: Machine learning insights into vaccine adjuvants and immune outcomes
Source: Front Immunol. 2025 Oct 7;16:1654060. doi: 10.3389/fimmu.2025.1654060 (PMC12537785; doi:10.3389/fimmu.2025.1654060)
Supplement: Supplementary Table 1 — Information of non-human primates used for this study. [file DataSheet1.docx]

Supplementary Table 1

Information of non-human primates used for this study.


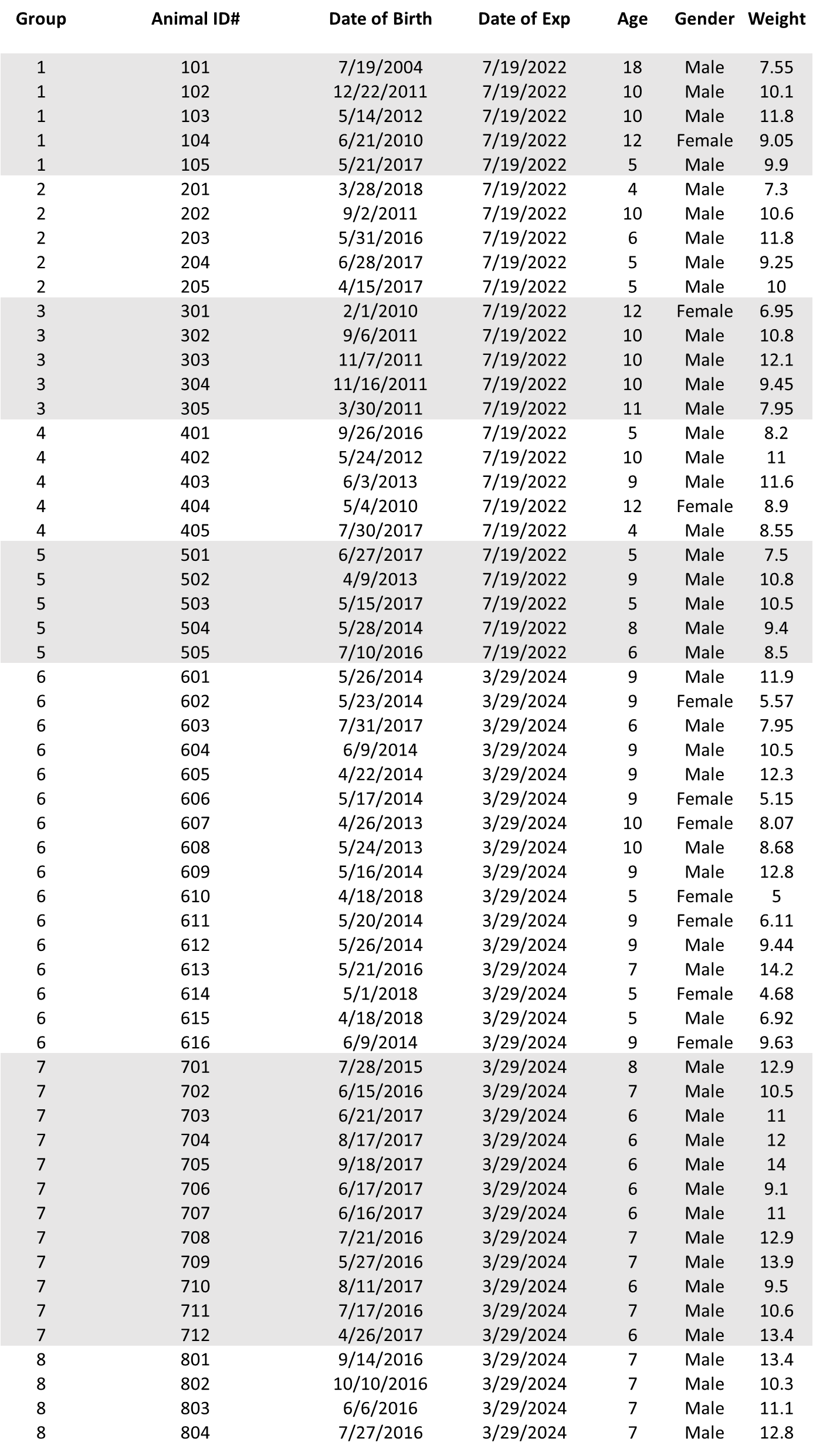


Supplementary Figure 1

Principal component analysis of gene expression post-dose: A principal component analysis (PCA) plot that illustrates the variance in gene expression between samples taken at two distinct time points: 1 day post-dose and 7 days post-dose. The PCA plot encapsulates the differences and similarities between the two time points, providing a visual representation of the variance in gene expression.

Supplementary Figure 2

Gene set enrichment analysis (GSEA) of adjuvant-related genes using Biomart and Ingenuity Pathway Analysis (IPA): The analysis provides insights into the biological pathways these genes are involved in, offering a comprehensive view of the immune response. GSEA with a different databank for understanding the genes influenced by the adjuvants. a) WikiPathways 2019 human, b) Reactome 2022, c) BioPlanet 2019, D) BioCarta 2016, and e) IPA. Black dotted lines on the figure indicate the p-value of 0.05.

Supplementary Figure 3


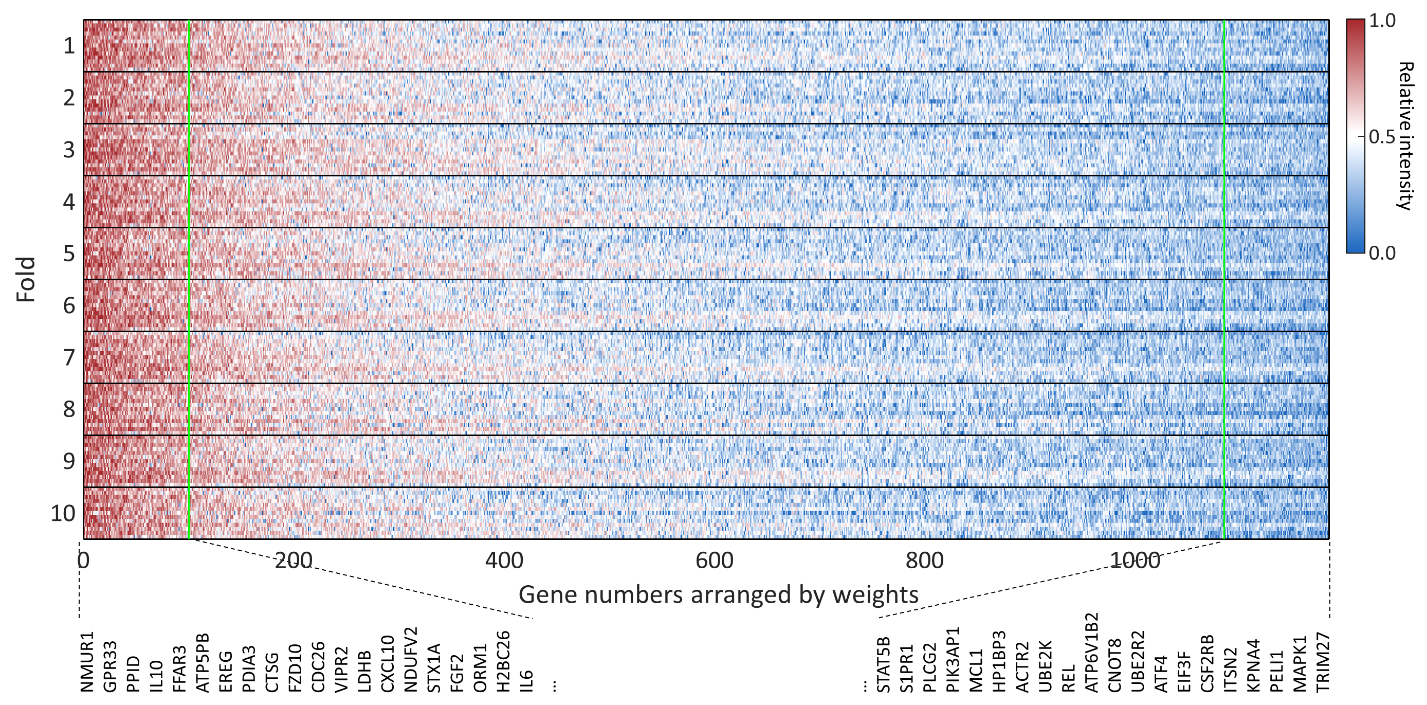


Weight analysis of trained deep learning model: a comprehensive analysis of the weights derived from the trained deep learning model. It emphasizes that some genes played a more pivotal role during the training process, thereby shedding light on the model's decision-making mechanism.

Supplementary Figure 4

Pearson correlation coefficient analysis of genes having high and low weights in the trained model: Pearson correlation coefficient analysis of the top 100 genes that were most and least influential during training. The analysis offers insights into the relationship between genes and their impact on the model's decision making. a) Genes obtained high weight during the training process for the classification task. Low coefficient values (0.35 with 0.13 standard deviation) with gentle slope between two different adjuvant groups are notable. This means that the same genes showed different expression levels. In other words, genes with high weights show different RNA expression patterns that can easily distinguish between two different groups. b) Genes obtained low eight during the training process for the classification task show a high coefficient value (0.50 with 0.18 standard deviation).

Supplementary Figure 5

Gene set enrichment analysis (GSEA) of highly weighted genes: This figure showcases a gene set enrichment analysis of the top 100 genes that had higher weights for the classification task. The analysis offers insights into the biological pathways these genes are involved in, thereby providing a deeper understanding of the adjuvant-induced changes. Also, the analysis can provide potential genes that should be more focused for studying adjuvant-specific mechanisms. GSEA with different datasets, including a) WikiPathways 2019 human, b) Reactome 2022, c) BioPlanet 2019, and D) BioCarta 2016, are shown.

Supplementary Figure 6

Predicted antibody titer using a deep learning model and its comparison with the ground truth value, measured antibody levels: Box-and-whisker plot illustrating the comparison between predicted antibody levels from deep learning model (orange boxes) and ground truth levels (blue boxes). The trends and mean antibody amounts between predictions and ground truth values show high alignment. The results demonstrate the capability of the deep learning model in understanding vaccine-induced RNA expression differences and the accuracy in predicting antibody levels.

Supplementary Figure 7


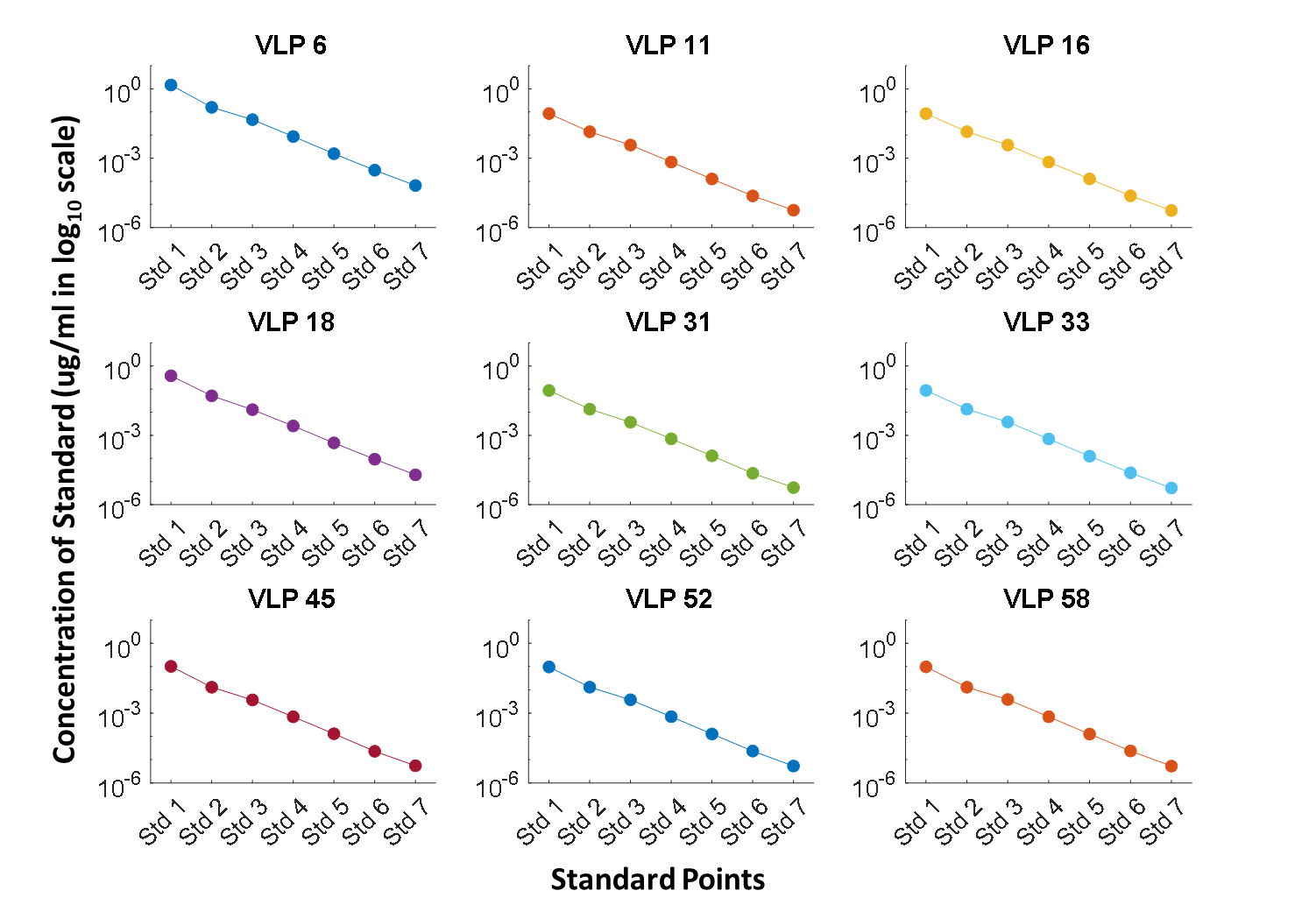


Standard curves for nine virus-like particle (VLP) types, displaying the measured signal for all seven standards (Std 1–Std 7). All points within the validated dynamic range are shown. A broad dynamic range indicates a reliable and proportional signal response across the entire concentration series. The y-axis is shown on a logarithmic scale to illustrate the full range of signal intensities.
